# IAMSST e Regurgitação Mitral Isquêmica: Incidência e Resultados Clínicos de Longo Prazo com Relação à Estratégia de Tratamento

**DOI:** 10.36660/abc.20240064

**Published:** 2024-11-22

**Authors:** Pooja Vyas, Radhakishan Dake, Kewal Kanabar, Iva Patel, Ashish Mishra, Vishal Sharma, Tirth Nathwani, Kunal Parwani, Mital Rathod

**Affiliations:** 1 Department of Cardiology U. N. Mehta Institute of Cardiology and Research Centre Civil Hospital Campus Gujarat Índia Department of Cardiology, U. N. Mehta Institute of Cardiology and Research Centre (UNMICRC), Civil Hospital Campus, Gujarat – Índia; 2 Champavati Medicare Heart and Critical Care Hospital Beed Maharashtra Índia Champavati Medicare Heart and Critical Care Hospital, Beed, Maharashtra – Índia; 3 Department of Research U. N. Mehta Institute of Cardiology and Research Centre Civil Hospital Campus Gujarat Índia Department of Research, U. N. Mehta Institute of Cardiology and Research Centre (UNMICRC), Civil Hospital Campus, Gujarat – Índia; 4 Interventional Cardiologist, Wockhardt Hospital Maharashtra Índia Interventional Cardiologist, Wockhardt Hospital, Evershine Rd, Maharashtra – Índia

**Keywords:** Insuficiência da Valva Mitral, Infarto do Miocárdio com Supradesnível do Segmento ST, Angioplastia Coronária com Balão

## Abstract

**Fundamento:**

O tratamento ideal para regurgitação mitral isquêmica (RMI) em pacientes com infarto do miocárdio sem supradesnivelamento do segmento ST (IAMSSST) é um tópico debatido.

**Objetivo:**

Avaliar o resultado a longo prazo em pacientes com IAMSSST e RMI, enfatizando particularmente a comparação de tratamentos naqueles com RM moderada a grave.

**Métodos:**

Inscrevemos pacientes com IAMSSST e classificamos regurgitação não/trivial a leve como RMI insignificante e regurgitação moderada a grave como RMI significativa. Além disso, pacientes com RMI substancial foram avaliados para desfechos clínicos de longo prazo com relação a diferentes estratégias de tratamento. Um teste foi considerado estatisticamente significativo com base no valor de probabilidade p<0,05.

**Resultados:**

De um total de 4.189 pacientes com IAMSSST, RMI significativa foi encontrada em 7,21% dos pacientes. Um número significativamente maior de pacientes com morte (1,21% vs. 13,24%, p<0,0001), choque cardiogênico (0,46% vs. 13,24%, p<0,0001) e insuficiência cardíaca (1,03% vs. 11,59%, p<0,0001) foram encontrados durante a hospitalização em pacientes com RMI significativa. Em um acompanhamento de 2 anos, uma taxa de evento maior foi observada no grupo RMI significativa. Pacientes com RMI significativa revascularizados por intervenção coronária percutânea (ICP), cirurgia de revascularização miocárdica (CRM) ou cirurgia CRM+ válvula mitral (VM) apresentaram melhora substancial no grau de RM (32,65% vs. 6% vs. 16,98%, p<0,0001) e fração de ejeção do ventrículo esquerdo (FEVE) (27,55% vs. 1% vs. 1,89%, p<0,0001) em 1 ano de acompanhamento e resultados significativamente melhores foram identificados em comparação ao grupo de revascularização recusada e tratamento médico com (-5,10% vs. 15% vs. 13,21%, p=0,04) mortalidade, (-33,67% vs. 61% vs. 73,58%, p<0,0001) readmissão e (- 15,31% vs. 27% vs. 33,96%, p=0,01) insuficiência cardíaca em 2 anos de acompanhamento.

**Conclusão:**

Maiores taxas de mortalidade e admissão foram observadas em pacientes com RMI significativa em comparação àqueles com RMI insignificante. Notavelmente, pacientes com RMI significativa que passaram por ICP, CRM ou cirurgia CRM+VM apresentaram melhores resultados em comparação com seus equivalentes não revascularizados.

## Destaque

• A presença de RM significativa em pacientes com IAMSSST leva a complicações importantes em curto e longo prazo.

• Taxas significativamente maiores de eventos cardiovasculares e readmissão foram associadas a pacientes com RM significativa.

• Entre os pacientes com RM significativa, aqueles que foram submetidos a qualquer procedimento de revascularização (ICP, CRM e CRM + cirurgia de VM) apresentaram uma taxa de mortalidade menor em comparação com aqueles que não foram submetidos a nenhum procedimento desse tipo.

## Graphical abstract

**Figura Central:**
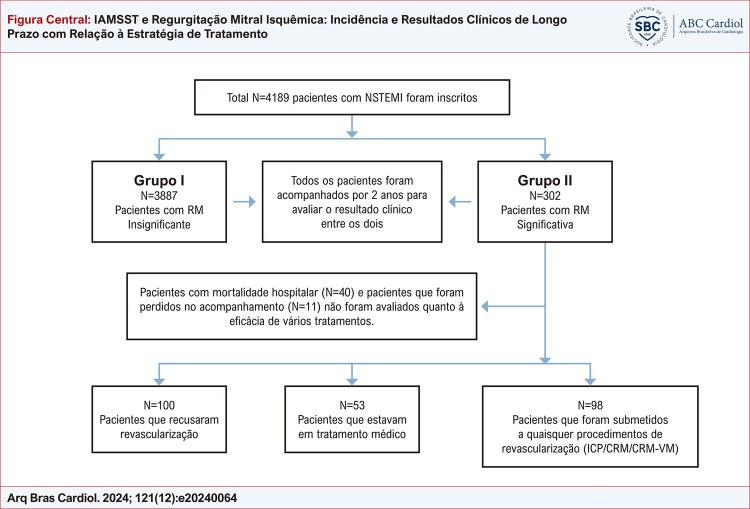
: IAMSST e Regurgitação Mitral Isquêmica: Incidência e Resultados Clínicos de Longo Prazo com Relação à Estratégia de Tratamento

## Introdução

O infarto do miocárdio sem supradesnivelamento do segmento ST (IAMSSST), caracterizado por dano miocárdico sem a elevação clássica do segmento ST, apresenta desafios distintos em seu diagnóstico e tratamento.^1^ As consequências do IAMSSST se estendem além do evento isquêmico imediato, muitas vezes contribuindo para uma cascata de complicações cardiovasculares.^2^ A prevalência de regurgitação mitral (RM) nessa coorte específica justifica um exame meticuloso, pois introduz uma camada de complexidade que pode influenciar a trajetória de recuperação e o prognóstico a longo prazo.

O desenvolvimento de RM em pacientes IAMSSST é multifatorial, envolvendo interações complexas entre vários processos patológicos. O desenvolvimento de RM no contexto de IAMSSST pode ser devido a patologias como disfunção muscular papilar isquêmica, ruptura muscular papilar, ruptura de cordas ou estiramento de cordas secundário à disfunção ventricular esquerda.^3^ A presença de RM hemodinamicamente significativa com IAMSSST pode levar à hipertensão venosa pulmonar, aumento do risco de insuficiência cardíaca, disfunção ventricular esquerda, aumento do risco de arritmias, piora de eventos isquêmicos e complicações pós-revascularização.^4^ A presença de RM em pacientes de IAMSSST leva a desafios adicionais ao prognóstico de longo prazo, afetando a função cardíaca, o risco de insuficiência cardíaca e a saúde cardiovascular geral. A identificação e o tratamento precoces de RM nesses pacientes podem impactar significativamente os resultados de longo prazo.

A gravidade da RM, seu impacto na função ventricular esquerda e o sucesso das intervenções moldam coletivamente a trajetória dos resultados dos pacientes em pacientes com IAMSSST. O objetivo deste estudo foi avaliar a prevalência de regurgitação mitral isquêmica (RMI). Ele analisou os resultados clínicos de longo prazo em pacientes, com foco específico na comparação daqueles com e sem RM significativa em relação à sua estratégia de revascularização.

## Métodos

### Desenho do estudo e população do estudo

Um total de 4189 pacientes diagnosticados com IAMSSST foram inscritos no presente estudo observacional de janeiro de 2016 a junho de 2021 em nosso centro de atendimento cardíaco terciário. Conduzimos um estudo de coorte retroprospectivo; os dados foram coletados retrospectivamente do ano de 2015 a 2018 e prospectivamente de 2019 a 2021 e acompanhados por dois anos. O estudo foi aprovado pelo comitê de ética institucional (UNMICRC/CARDIO/2019/14). O consentimento informado foi obtido de todos os participantes. No presente estudo, não contamos com a ajuda de nenhuma ferramenta de inteligência artificial.

Os dados demográficos, clínicos, ecocardiográficos e de desfecho hospitalar foram obtidos dos pacientes que foram diagnosticados com IAMSSST com base em biomarcadores cardíacos e diagnóstico de eletrocardiograma. Os dados retrospectivos durante a hospitalização e acompanhamento foram coletados do sistema EMR (*E-medical record*) do instituto. Pacientes com IAMCSST, angina instável, cardiomiopatia hipertrófica, doenças cardíacas valvulares ou estruturais, prolapsos mitrais e doenças cardíacas reumáticas, doença cardíaca congênita conhecida ou suspeita, anormalidades estruturais mitrais (valvulares/subvalvulares), doença primária de outra valva cardíaca, próteses mitrais, cirurgia de revascularização miocárdica (CRM) anterior ou intervenção coronária percutânea (ICP) anterior foram excluídos do estudo. Da coorte retrospectiva, foram excluídos os pacientes com dados incompletos e sem ecocardiografia de acompanhamento, em seguimento de 1 ano. Os pacientes foram agrupados de acordo com a gravidade da RMI avaliada na ecocardiografia 2D. A RMI foi categorizada como nenhuma/trivial e leve no grupo I de RMI insignificante e moderada a grave no grupo II de RMI significativa.^5^ Todos os pacientes no estudo foram submetidos à angiografia coronária. Além disso, os pacientes com RMI moderada a grave foram classificados em três subgrupos com base em sua abordagem de tratamento: aqueles que recusaram a revascularização, aqueles que foram aconselhados a tratamento médico e aqueles que foram submetidos a procedimentos de revascularização (ICP/CRM/cirurgia CRM-VM) e o resultado entre esses três grupos foi comparado. Pacientes com doença arterial coronária insignificante com obstrução coronária menor que 50% na estimativa visual da angiografia coronária, miocárdio não viável na cintilografia SPECT com Tc-99m sestamibi e a doença vascular de calibre estreito difusamente doente, não adequada para revascularização, foram indicadas para tratamento médico. Onze pacientes foram perdidos no acompanhamento em um grupo RMI significativo que não foram revascularizados.

### Acompanhamento e desfechos

Todos os pacientes do estudo foram monitorados por dois anos para avaliar eventos cardíacos adversos maiores (MACE). O acompanhamento telefônico foi feito para os pacientes que não compareceram ao departamento ambulatorial durante o período do estudo. Os principais MACE durante o hospital e nos períodos de acompanhamento foram definidos como morte cardiovascular, re-hospitalização, choque cardiogênico, acidente vascular cerebral cerebrovascular, insuficiência cardíaca e angina instável.

### Ecocardiografia

A ecocardiografia foi realizada usando um Philips Sonos 5500 equipado com sondas de 2,5-3,5 MHz. As medidas dos diâmetros atriais e ventriculares esquerdos foram feitas na vista paraesternal no modo M. Os cálculos da fração de ejeção foram realizados no modo 2D, utilizando as vistas apicais de 2 e 4 câmaras e empregando o método biplano de Simpson. Para avaliar o espessamento miocárdico, o ventrículo esquerdo foi dividido no modelo de 16 segmentos, seguindo as diretrizes recomendadas pela *American Society of Echocardiography*.^5^ A regurgitação miocárdica e sua gravidade foram avaliadas. Pacientes com ≥ 1 grau de melhora no RMI foram considerados como grau de RMI melhorado, e ≥ 1 grau piorado foi considerado como grau de RMI piorado no período de acompanhamento. FEVE no acompanhamento com aumento ≥5% foi considerada como melhora, e diminuição ≤5% foi considerada como piora no acompanhamento.

### Análise estatística

Variáveis de categorias foram expressas como frequências absolutas e relativas e comparadas usando análise qui-quadrado. Variáveis contínuas apresentaram distribuição normal, sendo representadas como média ± desvio padrão (DP) e comparadas usando um teste t de amostra independente. Testes de Shapiro-Wilk foram usados para verificar a normalidade. Variáveis que influenciam RMI significativo foram avaliadas usando análise de regressão logística. Um valor de probabilidade (valor p) menor que 0,05 foi considerado estatisticamente significativo. Pacientes com RMI significativo (grupo II) foram analisados posteriormente para desfecho no tempo de acompanhamento de acordo com o tratamento de escolha (procedimento vs. medicação) no momento em que foram incluídos, e pacientes com mortalidade hospitalar no grupo II não foram incluídos na análise de subgrupo. Todas as análises estatísticas foram realizadas usando o programa SPSS (*Statistical Package for the Social Science*) vs 20.

## Resultados

A Tabela 1 apresenta as características demográficas e clínicas basais comparadas entre dois grupos de RMI. Gênero feminino: presença de diabetes, pressão arterial sistólica baixa, FEVE reduzida < 40% (24,62% vs. 50,3%, p < 0,0001) e doença multiarterial foram encontrados significativamente maiores no grupo RMI moderada a grave (Grupo II) em comparação ao grupo RMI insignificante (Grupo I).

**Tabela 1:** – Características clínicas basais da população do estudo

	Grupo - I (RM insignificante) N=3887	Grupo- II (RM significativa) N=302	valor p
Idade	59,84±10,52	62,49±10,85	<0,0001
Fem.	901 (23,2%)	129 (42,7%)	<0,0001
Masc.	2986 (76,8%)	173 (57,3%)	
Diabetes	562 (14,5%)	105 (34,8%)	<0,0001
Hipertensão	1166 (29,99%)	97 (32,12%)	0,4784
Adição	619 (15,92%)	56 (18,54%)	0,2666
SCA antigo	1136 (29,2%)	84 (27,81%)	0,649
Frequência cardíaca	80,92±13,06	85,41±15,33	0,09
PAS	130,44±17,50	128,36±17,70	0,05
PAD	74,97±13,99	75,03±14,67	0,9430
**FEVE**			
FEVE≥50%)	1912 (49,19%)	39 (12,9%)	<0,0001
FEVE = 40-49%	1018 (26,19%)	111 (36,8%)	
FEVE <40%	957 (24,62%)	152 ( 50,3%)	
**Nº de embarcações bloqueadas**			
DVU	1768 (45,5%)	87 (28,81%)	<0,0001
DVD	1141 (29,35%)	98 (32,45%)	
DVT	978 (25,2%)	117 (38,74%)	

RM: regurgitação mitral; SCA: síndrome coronariana aguda; PAS: pressão arterial sistólica; PAD: pressão arterial diastólica; FEVE: fração de ejeção do ventrículo esquerdo; DSV: doença de vaso único; DVD: doença de vaso duplo; DVT: doença de vaso triplo.

A Tabela 2 mostra a análise de regressão logística univariada e multivariada para RMI significativa. Idade mais alta, presença de diabetes e FEVE mais baixa foram considerados preditores independentes de RM significativa.

**Tabela 2:** – Fatores associados à regurgitação mitral isquêmica significativa

	Univariada			Multivariado		
Parâmetros	OR	IC 95%	Valor-p	OR	IC 95%	Valor-p
**Fatores de risco**						
Idade	1.01	1-1,03	0,05	1.03	1,01-1,06	0,05
Fem.	1,89	1,25-2,86	0,003	1,70	0,75-3,82	0,202
Diabetes	3.15	2,45-4,07	<0,0001	2.27	1.01-5.17	0,05
Hipertensão	0,66	0,51-0,85	0,002	0,46	0,20-1,08	0,07
Tabagismo	0,63	0,37-1,07	0,09	0,52	0,18-1,41	0,197
**Parâmetros clínicos**						
Doença multiarterial	1,67	1,32-2,11	<0,0001	1,64	0,76-3,55	0,208
FEVE (%)	0,95	0,93-0,97	<0,0001	0,926	0,89-0,95	<0,0001

FEVE: fração de ejeção do ventrículo esquerdo; OR: razão de chances; IC: intervalo de confiança.

Pacientes com RMI significativa demonstraram uma taxa de mortalidade hospitalar notavelmente elevada, juntamente com uma maior ocorrência de choque cardiogênico e insuficiência cardíaca, em comparação com pacientes com RMI insignificante ao longo do curso da hospitalização. No acompanhamento de 1 e 2 anos, a mortalidade, a taxa de readmissão (e a incidência de insuficiência cardíaca) foram significativamente maiores em pacientes com RMI significativa (Tabela 3).

**Tabela 3:** – Comparação dos resultados clínicos hospitalares e de longo prazo

	Grupo- I N=3887	Grupo- II N=302	Valor-p
**Eventos clínicos adversos graves intra-hospitalares N (%)**			
Morte	47 (1,21%)	40 (13,24%)	<0,0001
Choque cardiogênico	18 (0,46%)	10 (3,31%)	<0,0001
Acidente vascular cerebral	01 (0,10%)	00	0,10
Insuficiência cardíaca	40 (1,03%)	35 (11,59%)	<0,0001
**Eventos clínicos adversos graves em 1 ano N (%)**			
Morte	176 (4,53%)	58 (19,21%)	<0,0001
Readmissão	682 (17,86%)	87 (34,66%)	<0,0001
Insuficiência cardíaca	190 (4,89%)	71 (23,51%)	<0,0001
Angina instável	71(1,85%)	5 (1,99%)	0,9925
**Eventos clínicos adversos graves em 2 anos N (%)**			
Morte	243 (6,25%)	67 (22,18%)	<0,0001
Readmissão	943 (24,26%)	133 (44,04%)	<0,0001
Insuficiência cardíaca	375 (9,65%)	95 (31,46%)	<0,0001
Angina instável	85 (2,17%)	09 (2,98%)	0,4870

Pacientes avaliados para fração de ejeção do ventrículo esquerdo e grau de RMI na ecocardiografia em 1 ano de acompanhamento. Um número significativamente maior de pacientes revascularizados teve uma redução na gravidade da RM em 1 ano de acompanhamento em comparação com pacientes em tratamento médico e pacientes que recusaram a revascularização. A FEVE foi significativamente melhorada em pacientes submetidos à revascularização (Tabela 4).

**Tabela 4:** – Resultado ecocardiográfico no seguimento de 1 ano

	Revascularização recusada (N=100)	Manejo médico (N=53)	Revascularizado (N=98)	Valor-p
**Grau RMI**				
Melhorou	06 (6%)	09 (16,98%)	32 (32,65%)	<0,0001
Semelhante	92 (92%)	44 (83%)	66 (67,35%)	0,0001
Piorou	02 (2%)	00	00	0,2182
**FEVE (%)**				
Melhorou	01 (1%)	01 (1,89%)	27 (27,55%)	<0,0001
Semelhante	87 (87%)	49 (92,45%)	68 (69,38%)	0,0004
Piorou	12 (12%)	03 (5,66%)	03 (3,06%)	0,04

RMI: regurgitação mitral isquêmica; FEVE: fração de ejeção do ventrículo esquerdo.

A Tabela 5 apresenta os eventos cardiovasculares em 1 e 2 anos de acompanhamento em pacientes com RM significativa e agrupados de acordo com o tratamento que receberam. No acompanhamento de dois anos, as taxas de mortalidade foram de 15%, 13,21% e 5,10% com p=0,04, enquanto as taxas de readmissão foram de 61%, 73,58% e 33,67% com p<0,0001, e as taxas de insuficiência cardíaca foram de 27%, 33,96% e 15,31% com p=0,001, em pacientes que recusaram a revascularização, pacientes em tratamento médico e pacientes que foram submetidos à revascularização, respectivamente.

**Tabela 5:** – Resultado clínico em 1 e 2 anos de acompanhamento de acordo com a estratégia de tratamento

	Revascularização recusada (N=100)	Manejo médico (N=53)	Revascularizado (N=98)	Valor p
**Eventos clínicos adversos graves em 1 ano N (%)**				
Morte	10 (10%)	05 (9,43%)	03 (3,06%)	0,1290
Readmissão	40 (40%)	23 (43,39%)	24 (24,49%)	0,02
Insuficiência cardíaca	19 (19%)	08 (15,1%)	9 (9,18%)	0,1414
**Eventos clínicos adversos graves em 2 anos N (%)**				
Morte	15 (15%)	07 (13,21%)	05 (5,10%)	0,04
Readmissão	61 (61%)	39 (73,58%)	33 (33,67%)	<0,0001
Insuficiência cardíaca	27 (27%)	18 (33,96%)	15 (15,31%)	0,001

A Tabela 6 apresenta os preditores de MACE. As probabilidades não ajustadas de RMI significativo mostraram-se significativamente altas (OR= 2,35), que diminuíram quando ajustadas com idade, diabetes, % FEVE e doença multiarterial (OR=1,09), no entanto, permaneceram significativas.

**Tabela 6:** – Preditor de MACE

Variáveis	Razão de chances (OR)	IC 95%	Valor-p
RMI significativa (não ajustada)	2,35	1,55-3,67	<0,001
RMI significativa ajustada para idade	1,99	1,05-2,93	<0,02
RMI significativa ajustada para diabetes	1,56	1,01-1,91	<0,02
RMI significativa ajustada para % FEVE	1,60	1.03-2.30	<0,01
RMI significativa ajustada para doença multiarterial	1.12	1,10-2,48	<0,01
RMI significativa ajustada para idade, diabetes, % FEVE, doença multiarterial	1.09	1,02-1,98	<0,04

RMI: regurgitação mitral isquêmica; FEVE: fração de ejeção do ventrículo esquerdo.

A Tabela 7 representa as características basais dos três grupos de acordo com a estratégia de tratamento. Pacientes com diabetes, dependência, síndrome coronariana aguda (SCA) antiga e doença multiarterial foram significativamente maiores em pacientes encaminhados para tratamento médico, enquanto os valores de FEVE e pressão arterial sistólica foram encontrados como menores em comparação com revascularização recusada e revascularização.

**Tabela 7:** – Comparação das características basais da população segundo a estratégia de manejo

	Revascularização recusada (N=111)	Manejo médico (N=53)	Revascularizado (N=138)	Valor-p
Idade	60,72±8,91	66,01±7,01	61,54±7,95	<0,0001
Fem.	49 (44,1%)	21 (39,6%)	59 (42,8%)	0,86
Masc.	62 (55,9%)	32 (60,4%)	79 (57,2%)	
Diabetes	30 (27,03%)	32 (60,38%)	43 (31,14%)	<0,0001
Hipertensão	38 (34,2%)	11 (20,8%)	39 (28,3%)	0,20
Adição	17 (15,32%)	28 (52,83%)	11 (8%)	<0,0001
SCA antigo	20 (18,02%)	35 (66,04%)	29 (21,01%)	<0,0001
Frequência cardíaca	84,23±10,23	88,19±11,39	85,69±9,96	0,07
PAS	127,05±22,23	120,60±20,96	129,62±22,30	0,04
PAD	74,99±15,60	72,13±12,96	75,99±15,54	0,29
FEVE	35,85±10,56	32,40±11,78	39,48±11,01	<0,0001
**Nº de vasos bloqueados**				
DVU	39 (35,13%)	03 (5,66%)	45 (32,61%)	<0,0001
DVD	35 (31,53%)	05 (9,43%)	58 (42,03%)	
DVT	37 (33,33%)	45 (84,90%)	35 (25,36%)	

SCA: síndrome coronariana aguda; PAS: pressão arterial sistólica; PAD: pressão arterial diastólica; FEVE: fração de ejeção do ventrículo esquerdo; DVU: doença de vaso único; DVD: doença de vaso duplo; DVT: doença de vaso triplo.

## Discussão

No estudo observacional atual, RMI significativa foi observada em 7,21% dos pacientes com IAMSSST. Idade mais avançada, presença de diabetes e menor % de FEVE foram associados a RMI significativa em pacientes com IAMSSST. Durante a hospitalização, incidências significativamente maiores de morte (1,21% vs. 13,24%), choque cardiogênico (0,46% vs. 3,31%) e insuficiência cardíaca (1,03% vs. 11,59%) foram encontradas em pacientes com RMI significativa. Na análise de resultados de subgrupo em pacientes com RM significativa, os pacientes revascularizados por qualquer ICP, CRM e CRM-RVM apresentaram MACE significativamente menores e FEVE e grau de RM melhorados em comparação aos pacientes tratados com tratamento médico e pacientes que recusaram a revascularização. Os pacientes que recusaram a revascularização apresentaram prognóstico significativamente ruim no acompanhamento. Após dois anos de acompanhamento, a incidência de mortalidade, readmissão e insuficiência cardíaca foi significativamente maior entre os pacientes que recusaram a revascularização e os pacientes com tratamento médico.

Investigações anteriores haviam encontrado uma taxa de incidência de 29,4%^6^ para RM em pacientes com IAM e 40,08%^7^ em pacientes com IAMSSST. Ao mesmo tempo, a incidência de RMI significativa foi relatada em 1,19%.^8^ e 21,73%.^9^ em pacientes com IAMSSST. Villanueva et al. 9 estudo com 21,73% de incidência para RMI significativa foi maior; isso pode ser devido à pesquisa ter incluído apenas pacientes mais velhos ≥80 anos de idade. No presente estudo, observamos 7,21% de incidência de RMI significativa entre pacientes IAMSSST.

Em indivíduos com RMI, passar por tratamento com ICP, CRM ou uma combinação de CRM e cirurgia de VM está ligado a melhores resultados de sobrevivência em comparação aos resultados associados apenas à terapia médica.^10^ A RM isquêmica causa alterações na estrutura e função do ventrículo esquerdo devido à doença cardíaca isquêmica, o que piora o prognóstico em pacientes com IM agudo. Cerca de 50% dos pacientes diagnosticados com insuficiência cardíaca congestiva e 20% dos pacientes com infarto agudo do miocárdio documentaram RMI.^4^ A sobrecarga crônica de volume causada pela RMI desencadeia a remodelação ventricular esquerda, alterando a estrutura e a função do coração. Esse processo de remodelação pode levar a mais disfunção cardíaca, aumentando a suscetibilidade a eventos cardiovasculares adversos. Esses sintomas, quando não tratados, podem contribuir para um declínio na saúde geral e um risco aumentado de mortalidade.

Um estudo feito por James et al. relatou que as taxas de mortalidade foram de 24% em 30 dias (IC de 95%, 12% a 36%), 42% em 6 meses (IC de 28% a 56%), 52% em 1 ano (IC de 38% a 66%) em pacientes com RMI isquêmica aguda moderadamente grave a grave.^11^ Uma análise multivariável sugeriu que a RM moderadamente grave ou grave pode ser um potencial preditor independente de mortalidade (p=0,06).^11^ Em uma análise multivariada após o ajuste para características basais, incluindo idade e FE, o risco relativo para mortalidade por todas as causas e cardíaca foi independentemente associado à presença de RMI com RR = 1,88 (p=0,003) e RR = 1,83 (p=0,014), respectivamente.^12^ Estudo prospectivo anterior envolvendo indivíduos com disfunção ventricular esquerda isquêmica crônica (fração de ejeção ≤ 45%) e pelo menos regurgitação mitral funcional (RMI) leve, o estudo descobriu que a gravidade da RMI em condições basais (ERO ≥ 20 mm^2^) previu independentemente apenas a morte cardíaca.^13^ No presente estudo, observamos 13,24% de morte hospitalar, 3,31% de choque cardiogênico e 11,59% de participantes com insuficiência cardíaca em pacientes com RMI significativa, o que foi significativamente maior em comparação com pacientes com RMI insignificante. No acompanhamento de 2 anos, a taxa de mortalidade foi de 22,18%, e 31,46% dos casos de insuficiência cardíaca foram observados em pacientes com RMI significativa. Entre os pacientes com RMI significativa, uma taxa de readmissão de 44,04% foi observada em 2 anos. Além disso, categorizamos apenas os pacientes com RMI significativa de acordo com as escolhas de tratamento que fizeram durante o tempo de admissão e comparamos os resultados no tempo de acompanhamento; descobrimos que os pacientes com revascularização (ICP ou CRM ou CRM + RVM) apresentaram melhor grau de RMI e FEVE no tempo de acompanhamento em comparação com os pacientes que recusaram a revascularização e os pacientes com tratamento médico. Mesmo esses pacientes apresentaram maior incidência de morte, insuficiência cardíaca e readmissão em 2 anos de acompanhamento. Consistente com o estudo atual, descobertas anteriores^10^ indicaram que os pacientes que receberam tratamento de qualquer ICP, CRM ou CRM + cirurgia de VM demonstraram maior sobrevida em comparação com aqueles tratados com abordagens médicas. Ambos os ensaios CTSN, um envolvendo RMI moderado e o outro envolvendo pacientes com RMI grave, não mostraram diferença entre a doença coronária-CRM e CRM mais reparo da válvula mitral (RVM) em termos de remodelação reversa do ventrículo esquerdo ou sobrevivência na marca de dois anos entre pacientes com RMI moderada e grave, o que sugere que não haverá benefícios significativos para pacientes que optarem por se submeter à RVM. Embora o RVM tenha oferecido uma correção mais duradoura da RM, não demonstrou um aumento significativo na sobrevivência ou uma redução em eventos adversos gerais ou readmissões.^14,15^ Dados relatados recentemente indicam que tratamento precoce de RMI concomitante à revascularização coronária, seja por CRM ou ICP, melhora a sobrevida a longo prazo em comparação à cirurgia de VM tardia após revascularização coronária. No entanto, pacientes com revascularização coronária prévia geralmente apresentam melhores resultados com ICP em comparação com CRM.^16^ Esses estudos, juntamente com a pesquisa atual, destacam a importância de considerar tratamentos de revascularização para pacientes com RMI grave com base na gravidade da doença e nas recomendações de profissionais de saúde, em vez de optar apenas pelo tratamento médico. No entanto, o sucesso das intervenções depende de vários fatores-chave, incluindo a gravidade da RMI, a extensão do infarto do miocárdio, a FEVE, a idade do paciente, a presença de comorbidades e a resposta específica do paciente. Ao aliviar a carga isquêmica e restaurar o fluxo sanguíneo normal, a ICP e a CRM demonstram estratégias valiosas para melhorar a função cardíaca e potencialmente melhorar a RM.

Ao navegar pelas complexidades do gerenciamento de RMI, a tomada de decisão personalizada e baseada em evidências é primordial. A integração dessas modalidades de tratamento, adaptadas às características individuais do paciente, contribui para uma melhor sobrevivência, melhor qualidade de vida e uma redução na carga geral de morbidade cardiovascular. À medida que a pesquisa avança e nossa compreensão de RMI se aprofunda, os esforços contínuos para refinar e adaptar as estratégias de tratamento prometem melhorar ainda mais os resultados para indivíduos que lutam com essa desafiadora condição cardiovascular.

### Limitação

Foi desafiador eliminar a variação interobservador na avaliação da RM. É importante notar que os achados do nosso estudo podem ser aplicáveis somente a pacientes com IAMSSST, mas generalizar para todos os pacientes com IAM pode não ser garantido. Os pacientes em tratamento médico eram mais graves (tinham idade mais avançada, diabete, dependência, SCA antiga, doença de três vasos e menor FEVE). Esses fatores podem contribuir para piores resultados independentemente da revascularização. Por isso, este estudo não permitiu estabelecer que a revascularização é a melhor opção de tratamento para RMI significativa após IAMSSST.

## Conclusão

Concluindo, abordar a RMI é um desafio multifacetado com implicações profundas para os resultados dos pacientes. O impacto prejudicial da RMI na mortalidade e morbidade ressalta a necessidade crítica de intervenções eficazes. Tratamentos como ICP, CRM e a abordagem abrangente CRM + RVM demonstraram seu potencial para mitigar os efeitos adversos da RMI.
