# Supplementary material for: IAMSST e Regurgitação Mitral Isquêmica: Incidência e Resultados Clínicos de Longo Prazo com Relação à Estratégia de Tratamento
Source: Arq Bras Cardiol. 2024 Nov 22;121(12):e20240064. [Article in Portuguese] doi: 10.36660/abc.20240064 (PMC11634318; doi:10.36660/abc.20240064)
Supplement: Translation

# Translation: NSTEMI and Ischemic Mitral Regurgitation: Incidence and Long-Term Clinical Outcomes with Respect to Management Strategy

**DOI:** 10.36660/abc.20240064i

**Authors:** Pooja Vyas, Radhakishan Dake, Kewal Kanabar, Iva Patel, Ashish Mishra, Vishal Sharma, Tirth Nathwani, Kunal Parwani, Mital Rathod

**Affiliations:** 1 India Department of Cardiology, U. N. Mehta Institute of Cardiology and Research Centre (UNMICRC), Civil Hospital Campus, Gujarat – India; 2 India Champavati Medicare Heart and Critical Care Hospital, Beed, Maharashtra – India; 3 India Department of Research, U. N. Mehta Institute of Cardiology and Research Centre (UNMICRC), Civil Hospital Campus, Gujarat – India; 4 India Interventional Cardiologist, Wockhardt Hospital, Evershine Rd, Maharashtra – India

**Keywords:** Mitral Valve Insufficiency, ST Elevation Myocardial Infarction, Coronary Angioplasty Balloon

## Abstract

**Background:**

The optimal treatment for ischemic mitral regurgitation (IMR) in patients of non-ST elevation myocardial infarction (NSTEMI) is a debated topic.

**Objective:**

To evaluate the long term outcome on patients with NSTEMI and IMR, particularly emphasizing the comparison of treatments in those with moderate to severe MR.

**Methods:**

We enrolled patients with NSTEMI and classified non/trivial to mild regurgitation as insignificant IMR and moderate to severe regurgitation as significant IMR. Furthermore, patients with substantial IMR were assessed for long-term clinical outcomes with respect to different management strategies. A test was considered statistically significant based on the probability value p<0.05.

**Results:**

From a total of 4,189 patients of NSTEMI, significant IMR was found in 7.21% of patients. A significantly higher number of patients with death (1.21% vs. 13.24%, p<0.0001), cardiogenic shock (0.46% vs. 13.24%, p<0.0001) and heart failure (1.03% vs. 11.59%, p<0.0001) were found during hospitalization in patients with significant IMR. At a 2-year follow-up, a higher event rate was observed in the significant IMR group. Patients with significant IMR re-vascularized either by percutaneous coronary intervention (PCI), coronary artery bypass grafting (CABG), or CABG+ mitral valve (MV) surgery showed substantial improvement in MR grade ( 32.65% vs. 6% vs. 16.98%, p<0.0001) and LVEF (27.55% vs. 1% vs. 1.89%, p<0.0001) at 1 year follow up and significantly improved outcomes were identified compared to refused revascularization and medical management group with (-5.10% vs. 15% vs. 13.21%, p=0.04) mortality, (-33.67% vs. 61% vs. 73.58%, p<0.0001) readmission, and (-15.31% vs. 27% vs. 33.96%, p=0.01) heart failure at 2 years follow up.

**Conclusion:**

Higher mortality and admission rates were observed in patients with significant IMR compared to those with in-significant IMR. Notably, significant IMR patients who underwent PCI, CABG, or CABG+MV surgery showed improved outcomes compared to non-revascularized counterparts.

## Highlight

•The presence of significant MR in patients with NSTEMI leads to major complications in the short and long term.

•Significantly higher cardiovascular events and readmission rate was associated with patients with significant MR.

•Among patients with significant MR, those who underwent any revascularization procedure (PCI, CABG, and CABG+ MV surgery) exhibited a lower mortality rate in comparison to those who did not undergo any such procedure.

## Introduction

Non-ST elevation myocardial infarction (NSTEMI), characterized by myocardial damage without the classic ST-segment elevation, poses distinct challenges in its diagnosis and management.^1^ The aftermath of NSTEMI extends beyond the immediate ischemic event, often contributing to a cascade of cardiovascular complications.^2^ The prevalence of MR in this specific cohort warrants meticulous examination, as it introduces a layer of complexity that can influence the trajectory of recovery and long-term prognosis.

The development of Mitral regurgitation (MR) in patients with NSTEMI is multifactorial, involving complex interactions between various pathological processes. Development of MR in the context of NSTEMI could be due to pathologies like ischemic papillary muscle dysfunction, papillary muscle rupture, chordal rupture, or chordal stretching secondary to left ventricular dysfunction.^3^ The presence of hemodynamically significant MR with NSTEMI can lead to pulmonary venous hypertension, increased risk of heart failure, left ventricular dysfunction, increased risk of arrhythmias, worsening of ischemic events, and complications post-revascularization.^4^The presence of Mitral Regurgitation in patients of NSTEMI leads to additional challenges to the long-term prognosis, affecting cardiac function, heart failure risk, and overall cardiovascular health. Early identification and management of MR in these patients can significantly impact long-term outcomes.

The severity of MR, its impact on left ventricular function, and the success of interventions collectively shape the trajectory of patients’ outcomes in patients of NSTEMI. The objective of this study was to assess the prevalence of ischemic mitral regurgitation (IMR). It analyzed the long-term clinical outcomes in patients, with a specific focus on comparing those with and without significant mitral regurgitation in relation to their revascularization strategy.

## Methods

### Study design and study population

A total of 4189 patients diagnosed with NSTEMI were enrolled in the present observational study from January- 2016 to June 2021 at our tertiary cardiac care center. We conducted a retro-prospective cohort study; data were taken retrospectively from the year 2015 to 2018 and prospectively from 2019 to 2021 and followed up for two years. The study was approved by the institutional ethics committee (UNMICRC/CARDIO/2019/14). The informed consent was obtained from all participants. In the present study, we have not taken the help of any Artificial intelligence tools.

The demographic, clinical, echocardiographic, and in-hospital outcome data were taken from the patients who were diagnosed with NSTEMI based on cardiac biomarkers and electrocardiogram diagnosis. The retrospective data during hospitalization and follow-up was collected from the institute’s EMR (E-medical record) system. Patients with STEMI, unstable angina, hypertrophic cardiomyopathy, valvular or structural heart diseases, mitral prolapses and rheumatic heart diseases, known or suspected congenital heart disease, structural mitral abnormalities (valvular/subvalvular), primary disease of another cardiac valve, mitral prostheses, prior coronary artery bypass grafting (CABG) or prior percutaneous coronary intervention (PCI) were excluded from the study. From the retrospective cohort, patients with incomplete data and without follow-up echocardiography, at 1 year follow-up were excluded. Patients were grouped according to the severity of mitral regurgitation (IMR) assessed on 2D echocardiography. IMR was categorized as none/trivial and mild in group I of in-significant IMR and moderate to severe in group II of significant IMR.^5^All patients in the study underwent coronary angiography. Furthermore, the patients with moderate to severe mitral regurgitation (IMR) were classified into three subgroups based on their treatment approach: those who refused revascularization, those who were advised medical management, and those who underwent revascularization procedures (PCI/CABG/CABG-mitral valve [MV] surgery) and outcome between these three groups were compared. Patients with insignificant coronary artery disease with coronary obstruction less than 50% on visual estimation of coronary angiography, nonviable myocardium on Tc-99m sestamibi SPECT scan, and diffusely diseased narrow caliber vessel disease not suitable for revascularization were advised medical management. Eleven patients were lost to follow-up in a significant IMR group who were not re-vascularized.

### Follow-up and Endpoints

All study patients were monitored over two years to assess major adverse cardiac events (MACE). The telephonic follow-up was taken for the patients who did not attend the outpatient department during the study period. The major adverse cardiac events (MACE) during the hospital and at follow-up times were defined as cardiovascular death, re-hospitalization, cardiogenic shock, cerebrovascular stroke, heart failure, and unstable angina.

### Echocardiography

Echocardiography was conducted using a Philips Sonos 5500 equipped with 2.5-3.5 MHz probes. Measurements of left atrial and ventricular diameters were taken in the parasternal view on M mode. Ejection fraction calculations were performed in 2D mode, utilizing the apical 2- and 4-chamber views and employing the Simpson biplane method. To assess myocardial thickening, the left ventricle was divided into the 16-segment model, following the guidelines recommended by the American Society of Echocardiography.^5^ The assessment of myocardial regurgitation and its severity was evaluated. Patients with ≥ 1 grade improvement in IMR were considered as IMR grade improved, and ≥ 1 grade worsened was considered as worsened IMR grade on follow-up period. Left ventricular ejection fraction (LVEF) on follow-up with ≥5% increase was considered as improved, and ≤5% decrease was considered as worsened at follow-up.

### Statistical analysis

Categories variables were expressed as absolute and relative frequencies and compared using chi-square analysis. Continuous variables presented normal distribution, being represented as mean ± standard deviation (SD) and compared using an independent sample t-test. Shapiro-Wilk tests were used to check normality. Variables influencing significant IMR were assessed using logistic regression analysis. A probability value (p-value) less than 0.05 was considered statistically significant. Patients with significant IMR (group II) were further analyzed for outcome at follow-up time according to the treatment of choice (Procedure vs. medication) at the time they enrolled, and patients with in-hospital mortality in group II were not included in the sub-group analysis. All statistical analysis was carried out using the SPSS (Statistical Package for the Social Science) program vs 20.

## Results

Table 1 presents baseline demographic and clinical characteristics compared between two groups of IMR. Female gender -, presence of diabetes -, low systolic blood pressure, reduced LVEF<40% (24.62% vs. 50.3%, p<0.0001), and multivessel disease were found significantly higher in moderate to severe IMR group (Group-II) compared to insignificant IMR group (Group-I).

**Table 1:** – Baseline clinical characteristic of the study population

	Group –I (Insignificant MR) N=3887	Group- II (Significant MR) N=302	p-value
Age	59.84±10.52	62.49±10.85	<0.0001
Female	901 (23.2%)	129 (42.7%)	<0.0001
Male	2986 (76.8%)	173 (57.3%)	
Diabetes	562 (14.5%)	105 (34.8%)	<0.0001
Hypertension	1166 (29.99%)	97 (32.12%)	0.4784
Addiction	619 (15.92%)	56 (18.54%)	0.2666
Old ACS	1136 (29.2%)	84 (27.81%)	0.649
Heart rate	80.92±13.06	85.41±15.33	0.09
SBP	130.44±17.50	128.36±17.70	0.05
DBP	74.97±13.99	75.03±14.67	0.9430
**LVEF**			
LVEF≥50%)	1912 (49.19%)	39 (12.9%)	<0.0001
LVEF = 40-49%	1018 (26.19%)	111 (36.8%)	
LVEF <40%	957 (24.62%)	152 ( 50.3%)	
**No. of Vessels blocked**			
SVD	1768 (45.5%)	87 (28.81%)	<0.0001
DVD	1141 (29.35%)	98 (32.45%)	
DVT	978 (25.2%)	117 (38.74%)	

ACS: acute coronary syndrome; SBP: systolic blood pressure; DBP: diastolic blood pressure; LVEF: left ventricular ejection fraction; SVD: single vessel disease; DVD: double vessel disease; TVD: triple vessel disease.

Table 2 shows the univariate and multivariate logistic regression analysis for Significant IMR. Higher age, presence of diabetes, and lower LVEF were found to be independent predictors of significant Mitral regurgitation.

**Table 2:** – Factors associated with significant IMR

	Univariate			Multivariate		
Parameters	OR	95% CI	p-value	OR	95% CI	p-value
**Risk factors**						
Age	1.01	1-1.03	0.05	1.03	1.01-1.06	0.05
Female	1.89	1.25-2.86	0.003	1.70	0.75-3.82	0.202
Diabetes	3.15	2.45-4.07	<0.0001	2.27	1.01-5.17	0.05
Hypertension	0.66	0.51-0.85	0.002	0.46	0.20-1.08	0.07
Smoking	0.63	0.37-1.07	0.09	0.52	0.18-1.41	0.197
**Clinical parameters**						
Multivessel disease	1.67	1.32-2.11	<0.0001	1.64	0.76-3.55	0.208
LVEF (%)	0.95	0.93-0.97	<0.0001	0.926	0.89-0.95	<0.0001

LVEF: left ventricular ejection fraction; OR: odds ratio; CI: confidence interval.

Patients with significant IMR demonstrated a notably elevated in-hospital mortality rate, alongside a heightened occurrence of cardiogenic shock and heart failure, in comparison to patients with insignificant IMR throughout the course of hospitalization. At 1 year and 2 years follow-up, death, readmission rate (and incidence of heart failure were significantly higher in patients with significant IMR (Table 3).

**Table 3:** – Comparison of in-hospital and long-term clinical outcomes

	Group- I N=3887	Group- II N=302	p-value
**In-hospital major adverse clinical events N (%)**			
Death	47 (1.21%)	40 (13.24%)	<0.0001
Cardiogenic shock	18 (0.46%)	10 (3.31%)	<0.0001
Cerebrovascular Stroke	01 (0.10%)	00	0.10
Heart failure	40 (1.03%)	35 (11.59%)	<0.0001
**1 year major adverse clinical events N(%)**			
Death	176 (4.53%)	58 (19.21%)	<0.0001
Readmission	682 (17.86%)	87 (34.66%)	<0.0001
Heart failure	190 (4.89%)	71 (23.51%)	<0.0001
Unstable angina	71(1.85%)	5 (1.99%)	0.9925
**2 years major adverse clinical events N(%)**			
Death	243 (6.25%)	67 (22.18%)	<0.0001
Readmission	943 (24.26%)	133 (44.04%)	<0.0001
Heart failure	375 (9.65%)	95 (31.46%)	<0.0001
Unstable angina	85 (2.17%)	09 (2.98%)	0.4870

Patients evaluated for left ventricular ejection fraction and IMR grade on Echocardiography at 1 year follow up. A significantly higher number of revascularized patients had a reduction in MR severity at 1-year follow-up as compared to patients on medical management and patients who refused revascularization. LVEF was found to be significantly improved in patients who underwent revascularization (Table 4).

**Table 4:** – Echocardiographic outcome at 1-year follow-up

	Refused revascularization (N=100)	Medical management (N=53)	Revascularized (N=98)	p-Value
**IMR grade**				
Improved	06 (6%)	09 (16.98%)	32 (32.65%)	<0.0001
Similar	92 (92%)	44 (83%)	66 (67.35%)	0.0001
Worsened	02 (2%)	00	00	0.2182
**LVEF (%)**				
Improved	01 (1%)	01 (1.89%)	27 (27.55%)	<0.0001
Similar	87 (87%)	49 (92.45%)	68 (69.38%)	0.0004
Worsened	12 (12%)	03 (5.66%)	03 (3.06%)	0.04

IMR: ischemic mitral regurgitation; LVEF: left ventricular ejection fraction.

Table 5 presents the cardiovascular events at 1 and 2 years of follow-up in patients with significant MR and grouped according to the treatment they received. At two-year follow-up, mortality rates were 15%, 13.21%, and 5.10% with p=0.04, while readmission rates were 61%, 73.58%, and 33.67% with p<0.0001, and rates of heart failure were 27%, 33.96%, and 15.31% with p=0.001, in patients who refused revascularization, patients on medical management and patients who underwent revascularization respectively.

**Table 5:** – Clinical outcome at 1 and 2 years follow up according to management strategy

	Refused revascularization (N=100)	Medical management (N=53)	Revascularized (N=98)	p Value
**1 year major adverse clinical events N (%)**				
Death	10 (10%)	05 (9.43%)	03 (3.06%)	0.1290
2Readmission	40 (40%)	23 (43.39%)	24 (24.49%)	0.02
Heart failure	19 (19%)	08 (15.1%)	9 (9.18%)	0.1414
**2 years major adverse clinical events N (%)**				
Death	15 (15%)	07 (13.21%)	05 (5.10%)	0.04
Readmission	61 (61%)	39 (73.58%)	33 (33.67%)	<0.0001
Heart failure	27 (27%)	18 (33.96%)	15 (15.31%)	0.001

Table 6 presents the predictors of MACE. Unadjusted odds of significant IMR showed significantly high (OR= 2.35), which was decreased when adjusted with age, diabetes, LVEF%, and multivessel disease (OR=1.09) however, it remained significant.

**Table 6:** – Predictor of MACE

Variables	Odds Ratio (OR)	95% CI	p-value
Significant IMR (unadjusted)	2.35	1.55-3.67	<0.001
Significant IMR Adjusted for age	1.99	1.05-2.93	<0.02
Significant IMR Adjusted for Diabetes	1.56	1.01-1.91	<0.02
Significant IMR Adjusted for LVEF%	1.60	1.03-2.30	<0.01
Significant IMR Adjusted for Multivessel disease	1.12	1.10-2.48	<0.01
Significant IMR Adjusted for Age, Diabetes, LVEF%, Multivessel disease	1.09	1.02-1.98	<0.04

IMR: ischemic mitral regurgitation; LVEF: left ventricular ejection fraction.

Table 7 represents the baseline characteristics of the three groups according to the management strategy. Patients with diabetes, addiction, old ACS, and multivessel disease were significantly higher in patients referred for medical management, while the values of LVEF and SBP were found to be lower compared to refused revascularization and revascularization.

**Table 7:** – Comparison of Baseline characteristics of the population according to the management strategy

	Refused revascularization (N=111)	Medical management (N=53)	Revascularized (N=138)	p-value
Age	60.72±8.91	66.01±7.01	61.54±7.95	<0.0001
Female	49 (44.1%)	21 (39.6%)	59 (42.8%)	0.86
Male	62 (55.9%)	32 (60.4%)	79 (57.2%)	
Diabetes	30 (27.03%)	32 (60.38%)	43 (31.14%)	<0.0001
Hypertension	38 (34.2%)	11 (20.8%)	39 (28.3%)	0.20
Addiction	17 (15.32%)	28 (52.83%)	11 (8%)	<0.0001
Old ACS	20 (18.02%)	35 (66.04%)	29 (21.01%)	<0.0001
Heart rate	84.23±10.23	88.19±11.39	85.69±9.96	0.07
SBP	127.05±22.23	120.60±20.96	129.62±22.30	0.04
DBP	74.99±15.60	72.13±12.96	75.99±15.54	0.29
LVEF	35.85±10.56	32.40±11.78	39.48±11.01	<0.0001
**No. of Vessels blocked**				
SVD	39 (35.13%)	03 (5.66%)	45 (32.61%)	<0.0001
DVD	35 (31.53%)	05 (9.43%)	58 (42.03%)	
TVD	37 (33.33%)	45 (84.90%)	35 (25.36%)	

ACS: acute coronary syndrome; SBP: systolic blood pressure; DBP: diastolic blood pressure; LVEF: left ventricular ejection fraction; SVD: single vessel disease; DVD: double vessel disease; TVD: triple vessel disease.

## Discussion

In the current observational study, significant IMR was observed in 7.21% of non-S-T elevation myocardial infarction patients. Higher age, presence of diabetes, and lower LVEF% were found to be associated with significant IMR in NSTEMI patients. During hospitalization, significantly higher incidences of death (1.21% vs. 13.24%), cardiogenic shock (0.46% vs. 3.31%), and heart failure (1.03% vs. 11.59%) were found in patients with significant IMR. In the sub-group outcome analysis in patients with significant MR, patients revascularized by any PCI, CABG, and CABG- mitral valve repair (MVR) showed significantly lower major adverse cardiac events and improved LVEF and MR grade compared to patients treated with medical management and patients who refused revascularization. Patients who refused revascularization showed significantly poor prognosis on follow-up. At two years follow-up, the incidence of mortality, readmission, and heart failure were significantly higher among patients who refused revascularization and patients with medical management.

Earlier investigations had -found an incidence rate of 29.4%^6^ for MR- in AMI patients and 40.08%^7^in NSTEMI patients. At the same time, the incidence of significant IMR was reported at 1.19%.^8^ and 21.73%.^9^ in NSTEMI patients. In the Villanueva et al.^9^ study 21.73% incidence for significant IMR was higher; that might be due to the survey enrolling only older patients ≥80 years of age. In the present study, we observed 7.21% of significant IMR incidence among NSTEMI patients.

In individuals with IMR, undergoing treatment with PCI, CABG, or a combination of CABG and MV surgery is linked to enhanced survival outcomes compared to the outcomes associated with medical therapy alone.^10^Ischemic MR causes changes in left ventricular structure and function due to ischemic heart disease, which worsens the prognosis in acute MI patients. Around 50% of the patients diagnosed with congestive heart failure and 20% of patients with acute myocardial infarction documented IMR.^4^The chronic volume overload caused by IMR triggers left ventricular remodeling, altering the structure and function of the heart. This remodeling process can lead to further cardiac dysfunction, increasing the susceptibility to adverse cardiovascular events. These symptoms, when left unaddressed, can contribute to a decline in overall health and an increased risk of mortality.

A study done by James et al. reported the mortality rates to be 24% at 30 days (95% CI, 12% to 36%), 42% at 6 months (CI, 28% to 56%), 52% at 1 year (CI, 38% to 66%) in acute ischemic moderately severe to severe IMR patients.^11^ A multivariable analysis suggested that moderately severe or severe mitral regurgitation may be a potential independent predictor of mortality (p=0.06).^11^ In a multivariate analysis after adjusting for baseline characteristics including age and EF, the relative risk for both all-cause and cardiac mortality were independently associated with the presence of IMR with RR=1.88 (p=0.003) and RR=1.83 (p=0.014), respectively.^12^ Previous prospective study involving individuals with chronic ischemic left ventricular dysfunction (ejection fraction ≤45%) and at least mild functional mitral regurgitation (IMR), the study found that the severity of IMR under basal conditions (ERO ≥20 mm^2^) independently predicted only cardiac death.^13^ In the present study, we observed 13.24% in-hospital death, 3.31% cardiogenic shock, and 11.59% participants with heart failure in patients with significant IMR, which was significantly higher compared to patients with in-significant IMR. At 2-year follow-up, the mortality rate was 22.18%, and 31.46% heart failure cases were observed in patients with significant IMR. Among patients with significant IMR, a readmission rate of 44.04% was observed at 2 years. Further, we categorized only significant IMR patients according to the treatment choices they made during admission time and compared outcomes at follow-up time; we found patients with revascularization (PCI or CABG or CABG + MVR) had improved IMR grade and LVEF at follow-up time compared to patients who refused revascularization and patients with medical management. Even these patients showed a higher incidence of death, heart failure, and readmission at 2 years follow-up. Consistent with the current study, previous findings^10^ indicated that patients receiving treatment either of any PCI, CABG, or CABG + MV surgery demonstrated enhanced survival compared to those managed with medical approaches. Both CTSN trials, one involving moderate IMR and the other involving severe IMR patients, showed no difference between CABG and CABG plus MV repair in terms of left ventricular reverse remodeling or survival at the two-year mark among patients with moderate and severe IMR which suggest that there will be no significant benefits for patients who choose to undergo MVR. While mitral valve repair offered a more enduring correction of mitral regurgitation, it did not demonstrate a significant enhancement in survival or a reduction in overall adverse events or readmissions.^14,15^ Recently reported data indicates that early treatment of IMR concomitant to coronary revascularization, either by CABG or PCI, improves long-term survival compared to delayed MV surgery after coronary revascularization. However, patients with Prior coronary revascularization generally experience better outcomes with PCI compared to CABG.^16^ These studies, along with the current research, highlight the importance of considering revascularization treatments for patients with severe IMR based on the severity of the disease and the recommendations of healthcare professionals rather than opting for medical management alone. However, the success of interventions depends on several key factors, including the severity of IMR, the extent of myocardial infarction, LVEF, the patient’s age, the presence of comorbidities, and the patient specific response. By alleviating the ischemic burden and restoring normal blood flow, PCI and CABG demonstrate valuable strategies to improve cardiac function and potentially improve mitral regurgitation.

In navigating the complexities of IMR management, personalized and evidence-based decision-making is paramount. The integration of these treatment modalities, tailored to individual patient characteristics, contributes to enhanced survival, improved quality of life, and a reduction in the overall burden of cardiovascular morbidity. As research advances and our understanding of IMR deepens, continued efforts to refine and tailor treatment strategies hold the promise of further improving outcomes for individuals grappling with this challenging cardiovascular condition.

### Limitation

It was challenging to eliminate inter-observer variation in the assessment of mitral regurgitation (MR). It’s important to note that our study findings may be only applicable to non-ST-segment elevation myocardial infarction (NSTEMI) patients, but generalizing to all acute myocardial infarction (AMI) patients may not be warranted. The patients on the medical management were more severe (they had higher age, diabetes, addiction, old ACS, three-vessel disease, and less LVEF). These factors could contribute to worse outcomes independent of the revascularization. Because of this, this study did not allow to establish that revascularization is the better treatment option for significant IMR after NSTEMI.

## Conclusion

In conclusion, addressing IMR is a multifaceted challenge with profound implications for patient outcomes. The detrimental impact of IMR on mortality and morbidity underscores the critical need for effective interventions. Treatments such as PCI, CABG, and the comprehensive CABG + MVR approach have demonstrated their potential to mitigate the adverse effects of IMR.
